# Assessment and application of host-specific *Bacteroidales* genetic markers for microbial source tracking of river water in Japan

**DOI:** 10.1371/journal.pone.0207727

**Published:** 2018-11-16

**Authors:** Eiji Haramoto, Rui Osada

**Affiliations:** 1 Interdisciplinary Center for River Basin Environment, Graduate Faculty of Interdisciplinary Research, University of Yamanashi, Kofu, Yamanashi, Japan; 2 Department of Civil and Environmental Engineering, Faculty of Engineering, University of Yamanashi, Kofu, Yamanashi, Japan; Oklahoma State University, UNITED STATES

## Abstract

Microbial source tracking using host-specific microbial genetic markers is considered a promising approach to determine fecal contamination sources of aquatic environments. This study aimed to assess the application of previously developed host-specific *Bacteroidales* quantitative PCR assays to microbial source tracking of river water samples in Yamanashi Prefecture, Japan. Various types of fecal-source samples, such as raw sewage, secondary-treated sewage of a wastewater treatment plant, and cattle feces, were used for three human-, two ruminant- and two pig-specific *Bacteroidales* quantitative PCR assays. Our results demonstrated that BacHum, BacR and Pig2Bac assays as suitable human-, ruminant- and pig-specific assays, with an accuracy of 86%, 94% and 77%, respectively. These selected assays were used for microbial source tracking of 63 river water samples collected at nine sites in two river basins. From these sites, there were 48 (76%), 34 (54%) and 9 (14%) positive samples using the BacHum, BacR and Pig2Bac assays, respectively. These assays revealed the effects of humans and animals on fecal contamination of river water.

## Introduction

Microbial source tracking based on detection of host-specific *Bacteroidales* genetic markers is a promising approach to determine the sources of fecal contamination of aquatic environments because of their high abundance in both human and animal feces [1, 2]. Many PCR and quantitative PCR (qPCR) assays have been developed for host-specific detection of *Bacteroidales* genetic markers. However, such assays can cross react with feces from non-target hosts, which is probably attributed to the difference in bacterial community structures of feces across different geographical regions [3, 4].

Although findings from studies covering more than 10 countries in six continents established gold standard assays, samples used in these studies were from selected regions [3, 5]. Therefore, it is important to evaluate the performance of these developed assays using fecal-source samples prior to being applied to different regions [6, 7]. This approach will be useful even if limited numbers of fecal-source samples are available. For example, in our previous study conducted in Nepal, only 2–12 fecal-source samples were collected from each host type, such as humans, ruminants and pigs; however, the most suitable *Bacteroidales* qPCR assay was successfully selected among multiple published assays for each host type, and they were further used for microbial source tracking of groundwater samples [7].

In this study, seven *Bacteroidales* qPCR assays (three human-, two ruminant- and two pig-specific) were evaluated, using various types of fecal-source samples, in their application to microbial source tracking in Yamanashi Prefecture, Japan. Selected assays were then used to detect host-specific *Bacteroidales* genetic markers in 63 river water samples collected in two river basins and to determine their utility in microbial source tracking.

## Materials and methods

### Sample collection

Fecal-source and river water samples were collected in Yamanashi Prefecture. For human fecal-source samples, raw sewage and secondary-treated sewage were collected at a wastewater treatment plant nine times between August and December 2016 (n = 9 each), whereas effluent of a domestic wastewater treatment tank was collected twice in December 2016 (n = 2). For ruminant fecal-source samples, cattle feces were collected from three pens in a cattle farm five times between October and December 2016. Unfortunately, no pig fecal samples were obtained in this study. Therefore, river water samples were collected at a site located downstream of a pig farm’s discharge point between February and October 2014 (n = 17). River water samples were also collected at a site upstream of the discharge point between June and October 2014 (n = 9), which was used to assess the effect of pig farm wastewater on water quality of downstream river water.

During a four-month period between September and December 2016, a total of 63 river water samples were collected at nine sites in two river basins to perform microbial source tracking using the selected *Bacteroidales* qPCR assays. Nine river water samples were collected from three sites (S1–S3) in the upstream area of the Shiokawa River basin, where there were no anthropogenic pollution sources observed. S1 was located most upstream, followed by S2 and S3. Fifty-four river water samples were also collected from six sites (F1–F6) in the Fujikawa River basin. F1–F5, from upstream to downstream, were located along the Arakawa River, a tributary river of the Fujikawa River. No wastewater treatment plant but many domestic wastewater treatment tanks existed in the Arakawa River basin. F6 was located downstream of the Fujikawa River basin, receiving water from many tributaries, including the Arakawa River. Thus, this site was considered possibly impacted by human feces from wastewater treatment plants and domestic wastewater treatment tanks, as well as animal feces from livestock facilities and wild animals.

Water samples were collected in 1-l autoclaved plastic bottles, whereas cattle fecal samples were collected in 50-ml sterilized plastic tubes using disposable spoons. All samples were kept in a cooler bag with cooling materials and transported to the laboratory within several hours after sample collection. Sampling was conducted after getting permissions from managers of the wastewater treatment plant and owners of the domestic wastewater treatment tank and pig farm, whereas no permission was needed for the river water sampling. The water sampling did not involve endangered or protected species.

### Concentration of bacteria

The water sample (10 ml for raw sewage, 100 ml for secondary-treated sewage and effluent of the domestic wastewater treatment tank and 500 ml for river water at S1–S3 and F1–F6) was filtered through a mixed cellulose ester membrane (pore size, 0.22 μm; diameter, 47 mm; Merck Millipore, Billerica, USA). For river water samples collected upstream and downstream of the pig farm, 2 l of the sample was filtered through a 0.45-μm mixed cellulose ester membrane (diameter, 90 mm; Merck Millipore).

Subsequently, the membrane was placed into a 50-ml plastic tube containing 10 ml of elution buffer (0.2-g/l Na_4_P_2_O_7_ 10H_2_O, 0.3 g/l C_10_H_13_N_2_O_8_Na_3_ 3H_2_O and 0.1 mL/l Tween 80), followed by vortexing for ~5 min. The eluate was recovered into another 50-ml tube. A similar procedure was repeated using 5 ml of elution buffer, and the eluate was transferred into the same tube. The tube was centrifuged at 2,000 × *g* for 10 min at 4°C, the supernatant was discarded, and the pellet was suspended with 1 ml of phosphate-buffered saline (PBS (–)) to obtain a bacteria-concentrated sample.

For cattle fecal samples, 0.4 g of the sample was mixed with 40 ml of PBS (–) to obtain a 1% fecal suspension, which was then vortexed for ~5 min. One milliliter of the suspension was centrifuged at 7000 × *g* for 10 min at 4°C, followed by removal of the supernatant and addition of 1 ml of PBS (–).

### Bacterial DNA extraction

Two hundred microlitres of the bacteria-concentrated sample was subjected to bacterial DNA extraction using a QIAamp DNA Mini Kit (QIAGEN, Hilden, Germany) and a QIAcube instrument (QIAGEN) to obtain a 200-μl DNA extract sample.

### qPCR for *Bacteroidales* genetic markers

Three human-specific (*gyr*B [8], BacHum [9] and HF183 TaqMan [10]), two ruminant-specific (BacR [11] and BacCow [9, 12]), and two pig-specific assays (Pig2Bac [13] and PF163-SYBR [7, 12, 14]) were tested in this study. qPCR was performed using a Thermal Cycler Dice Real Time System TP800 (Takara Bio, Kusatsu, Japan). In brief, for all assays, except for the PF163-SYBR assay, 2.5 μl of extracted DNA was mixed with 22.5 μl of qPCR mixture containing 12.5 μl of Probe qPCR Mix (Takara Bio), 1.0 μl each of forward and reverse primers (10 μmol/l), 1.0 μl of TaqMan (MGB) probe (5 μmol/l) and 7.0 μl of PCR-grade water. For the PF163-SYBR assay, 2.5 μl of extracted DNA was mixed with 22.5 μl of qPCR mixture containing 12.5 μl of SYBR Premix Ex Taq II (Tli RNaseH Plus) (Takara Bio), 1.0 μl each of forward and reverse primers (10 μmol/l), and 8.0 μl of PCR-grade water. For the TaqMan (MGB) probe-based assays, the reaction conditions were 95°C for 30 s, followed by 45 cycles at 95°C for 5 s and at 60°C for 30 s. For the PF163-SYBR assay, the reaction conditions were as follows: PCR amplification of 95°C for 30 s, 45 cycles at 95°C for 5 s, 55°C for 30 s and 72°C for 60 s, followed by a melting curve analysis of 95°C for 15 s, 60°C for 30 s and 95°C for 15 s.

Tenfold serial dilutions of synthesized plasmid DNA containing the amplification region sequence were used to prepare standard samples, whereas PCR-grade water was used for the negative control. Fecal-source and water samples were tested in duplicate, whereas standard samples and the negative control were tested in triplicate. A Thermal Cycler Dice Real Time System Software Version 5.11 (Takara Bio) was used for data analysis, where a cycle threshold value of 40 was set as a cut-off point. For the PF163-SYBR assay, a melting temperature of ~79°C was considered positive.

### Selection of *Bacteroidales* genetic markers

DNA extracted from the fecal-source samples were subjected to qPCR for three human-, two ruminant- and two pig-specific *Bacteroidales* qPCR assays. Sensitivity, specificity and accuracy were used to select the best performing assay [15]. Sensitivity was calculated as a ratio of the number of true-positive samples to the total number of true-positive and false-negative samples, specificity was calculated as a ratio of true-negative samples to the total number of true-negative and false-positive samples, and accuracy was calculated as a ratio of the sum of true-positive and true-negative samples to the total number of samples tested.

### Selected host-specific *Bacteroidales* qPCR assays for river water samples

The assay deemed best in each of the three host categories was used for microbial source tracking of the 63 river water samples collected in Yamanashi Prefecture.

### Detection of *Escherichia coli*

*E*. *coli* in the samples were measured by the most probable number (MPN) method using a Colilert (IDEXX Laboratories, Westbrook, USA) according to the manufacturer’s protocol. After a 24-h incubation at 37°C, the numbers of large and small wells with fluorescent blue colour under UV light exposure were counted, and the MPN value for *E*. *coli* was determined using an MPN generating software (IDEXX Laboratories).

### Statistical analysis

The χ^2^ test was used to assess the difference in performance between *Bacteroidales* qPCR assays, whereas the *t*-test was used to compare concentrations of the target markers between qPCR assays or samples. Microsoft Office Excel 2016 (Microsoft Corporation, Redmond, USA) was used to perform statistical analysis. *P* < 0.05 indicated statistical significance.

## Results and discussion

### Detection of *Bacteroidales* genetic markers in fecal-source samples

Table 1 summarizes the detection results of host-specific *Bacteroidales* genetic markers in fecal-source samples using seven different qPCR assays: three human-, two ruminant- and two pig-specific assays. As expected, all nine raw sewage samples of the wastewater treatment plant, serving as human fecal-source samples, were positive using the three human-specific assays, with high concentrations of 8.3 ± 0.2 (n = 9), 10.1 ± 0.2 (n = 9) and 9.5 ± 0.2 log copies/l (n = 9) by *gyrB*, BacHum and HF183-TaqMan assays, respectively. However, they were all positive using the ruminant-specific BacCow and pig-specific PF163-SYBR assays, with concentrations of 7.5 ± 0.3 (n = 9) and 7.3 ± 1.1 log copies/l (n = 9), respectively, and one sample was deemed positive using the ruminant-specific BacR assay, although the concentration was low (4.7 log copies/l). Similar non-specific detections, or false-positive results, were observed for other fecal-source samples not only from humans (secondary-treated sewage of the wastewater treatment plant and effluent of the domestic wastewater treatment tank) but also from cattle (cattle feces).

**Table 1:** Detection of host-specific *Bacteroidales* genetic markers in fecal-source samples.

Sample	No. of tested samples	No. of positive samples (% positive)						
		Human-specific assays			Ruminant-specific assays		Pig-specific assays	
		*gyr*B	BacHum	HF183-TaqMan	BacR	BacCow	Pig2Bac	PF163-SYBR
Raw sewage	9	9 (100)	9 (100)	9 (100)	1 (11)	9 (100)	0 (0)	9 (100)
Secondary-treated sewage	9	8 (89)	9 (100)	9 (100)	0 (0)	1 (11)	0 (0)	6 (67)
Effluent of a domestic wastewater treatment tank	2	2 (100)	2 (100)	2 (100)	0 (0)	2 (100)	0 (0)	2 (100)
Cattle feces	15	4 (27)	5 (33)	15 (100)	14 (93)	15 (100)	12 (80)	15 (100)
River water upstream of a pig farm	9	8 (89)	9 (100)	9 (100)	3 (33)	7 (78)	2 (22)	6 (67)
River water downstream of a pig farm	17	17 (100)	17 (100)	17 (100)	11 (65)	17 (100)	17 (100)	15 (88)

For this reason, three parameters, such as sensitivity, specificity and accuracy, were determined to find the best performing qPCR assay for each host type. As shown in Table 2, all three human-specific assays yielded high sensitivity (≥ 95%), whereas the specificity varied greatly from 0% to 73%, depending on the assay. As a result, the accuracy was calculated as 86% for both *gyrB* and BacHum assays and 57% for the HF183-TaqMan assay.

**Table 2:** Performance of host-specific *Bacteroidales* genetic markers.

Parameter	Percentage (no. of samples judged correctly/no. of tested samples)						
	Human-specific assays			Ruminant-specific assays		Pig-specific assays	
	*gyrB*	BacHum	HF183-TaqMan	BacR	BacCow	Pig2Bac	PF163-SYBR
Sensitivity	95 (19/20)	100 (20/20)	100 (20/20)	93 (14/15)	100 (15/15)	100 (17/17)	88 (15/17)
Specificity	73 (11/15)	67 (10/15)	0 (0/15)	95 (19/20)	40 (8/20)	66 (23/35)	9 (3/35)
Accuracy	86 (30/35)	86 (30/35)	57 (20/35)	94 (33/35)	66 (23/35)	77 (40/52)	35 (18/52)

For ruminant-specific assays, both assays tested showed high sensitivity (≥ 93%). However, the specificity of the BacR assay (95%) was significantly higher than that of the BacCow assay (40%) (χ^2^-test, *P* < 0.05), resulting in the significantly higher accuracy of the BacR assay (94%) (χ^2^-test, *P* < 0.05).

For the pig-specific assays, river water samples impacted by wastewater from a pig farm were considered as pig fecal-source samples. The Pig2Bac assay worked better than the PF163-SYBR assay, with a sensitivity of 100%, specificity of 66% and accuracy of 77%. River water samples were also collected from a site upstream of the pig farm, where 22% (2/9) of the samples were positive using the Pig2Bac assay. The concentrations of the Pig2Bac marker in the positive samples were significantly lower at the upstream site (2.5 ± 0.9 log copies/l; n = 2) than at the downstream site (4.3 ± 0.9 log copies/l; n = 17) (*t*-test, *P* < 0.05). This indicated that wastewater from the pig farm greatly affected the levels of pig fecal contamination at the downstream site, although the upstream site was also contaminated with pig feces.

Based on these results, BacR and Pig2Bac assays were more suitable to be used in the study area as ruminant- and pig-specific assays, respectively, which was completely consistent with the results in a previous study conducted in Nepal [7]. For human-specific assays, *gyrB* and BacHum assays similarly performed well with an accuracy of 86%, suggesting that either of the assays could be used in this study area. However, because higher concentrations of the BacHum marker were observed in the human fecal-source samples than the *gyrB* marker (*t*-test, *P* < 0.05), the BacHum assay was selected as the human-specific assay to increase the probability of detection in river water samples. The BacHum assay has been also judged best in other Asian countries, such as India [15] and Nepal [7], suggesting its applicability to the Asian region. The difference in the mean marker concentrations between the BacHum and *gyrB* assays was 1.8, 1.6 and 3.0 log for raw sewage, secondary-treated sewage and effluent of the domestic wastewater treatment tank, respectively. This observed difference may be partially explained by the lower copy number of the *gyrB* gene in bacterial cells [16] compared to the 16S rRNA gene, which is a target used in the BacHum assay [17].

All seven assays showed a sensitivity of ≥ 80%, a recommended acceptable value [18], whereas the specificity was lower than this value, even for the selected assays. This is partially because the limited number of non-target samples was tested. Thus, more fecal-source samples from other animal hosts, such as chicken or duck, will need to be included for assay validation and more accurate evaluation, which may increase specificity. Nevertheless, this study clearly highlighted the importance of validation using fecal-source samples prior to microbial source tracking of environmental water samples, as suggested previously [6, 7].

### Application of selected qPCR assays for river water samples

BacHum, BacR and Pig2Bac assays were further used for microbial source tracking of 63 river water samples collected from two river basins in Yamanashi Prefecture. As shown in Table 3, *E*. *coli*, a fecal indicator bacterium, was detected in all samples, including samples from the Shiokawa River basin, where there were no anthropogenic sources of fecal contamination. The mean concentration of *E*. *coli* was the highest at F6 (3.2 ± 0.7 log MPN/l), the downstream site of the Fujikawa River basin, which could be contaminated with not only effluent of wastewater treatment plants and domestic wastewater treatment tanks but also wastewater of livestock facilities and feces of wild animals.

**Table 3:** Detection of host-specific *Bacteroidales* genetic markers in river water samples.

Basin	Site	No. of tested samples	*E*. *coli*		BacHum		BacR		Pig2Bac	
			No. of positive samples (% positive)	Concentration (log MPN/l)	No. of positive samples (% positive)	Concentration (log copies/l)	No. of positive samples (% positive)	Concentration (log copies/l)	No. of positive samples (% positive)	Concentration (log copies/l)
Shiokawa River	S1	3	3 (100)	2.4 ± 0.7	0 (0%)	Not detected	3 (100%)	3.7 ± 0.2	0 (0%)	Not detected
	S2	3	3 (100)	2.4 ± 0.3	1 (33%)	4.4	3 (100%)	3.7 ± 0.2	0 (0%)	Not detected
	S3	3	3 (100)	2.3 ± 0.2	0 (0%)	Not detected	3 (100%)	4.0 ± 0.2	0 (0%)	Not detected
Fujikawa River	F1	9	9 (100)	2.9 ± 0.3	9 (100%)	5.6 ± 0.6	3 (33%)	3.9 ± 0.0	0 (0%)	Not detected
	F2	9	9 (100)	2.6 ±0.6	7 (78%)	4.9 ± 0.2	4 (44%)	4.0 ± 0.2	0 (0%)	Not detected
	F3	9	9 (100)	2.7 ± 0.5	9 (100%)	5.1 ± 0.2	5 (56%)	4.0 ± 0.5	1 (11%)	3.4
	F4	9	9 (100)	2.5 ± 0.5	5 (56%)	5.0 ± 0.6	3 (33%)	4.1 ± 0.2	0 (0%)	Not detected
	F5	9	9 (100)	2.6 ± 0.3	8 (89%)	4.9 ± 0.2	1 (11%)	4.3	0 (0%)	Not detected
	F6	9	9 (100)	3.2 ± 0.7	9 (100%)	6.3 ± 0.5	9 (100%)	4.2 ± 0.6	8 (89%)	4.1 ± 0.4

Human-specific BacHum marker was detected in 47 (87%) of 54 river water samples collected in the Fujikawa River basin, with concentrations of 4.4–7.1 log copies/l. The mean concentration of the marker was the highest at F6 (6.3 ± 0.5 log copies/l), a similar trend as to the *E*. *coli* detection. The BacHum marker was detected in only one (11%) of nine samples in the Shiokawa River basin, indicating lower levels of human fecal contamination.

Compared with the BacHum marker, the BacR marker was detected in 46% (25/54) of the Fujikawa River basin samples. Interestingly, all nine samples collected at three sites (S1–S3) in the Shiokawa River basin were positive for this marker, with concentrations of 3.4–4.1 log copies/l. Considering that there were no livestock facilities located upstream, these sites could have been contaminated with feces from wild ruminant animals, such as deer. This finding warrants further evaluation via performing nucleotide sequencing analysis of the 16S rRNA gene amplified in these samples.

The Pig2Bac marker was not detected in any of the nine samples collected from the Shiokawa River basin. Similarly, all samples at F1–F5 were negative for this marker, except for one sample collected at F3 in November 2016. By contrast, the Pig2Bac marker was detected in eight (89%) of nine samples at F6, with concentrations of 4.1 ± 0.4 log copies/l. These results could be explained by the land use data, which indicated that there are almost no pig farms found in the Arakawa River basin where F1–F5 are located, whereas F6 receives water not only from the Arakawa River but also from other tributaries, including those with many pig farms.

In summary, this study assessed the performance of previously developed host-specific *Bacteroidales* qPCR assays to identify suitable assays for microbial source tracking of river water samples. The selected assays successfully characterized sources of fecal contamination from river water samples, indicating that ruminant feces greatly impact upstream river water quality. Further studies are necessary to test qPCR assays targeting other possible hosts for better understanding of fecal contamination of aquatic environments.
